# Dose-Response Relationship Between Serum γ-Glutamyltransferase and Arterial Stiffness in Korean Adults: The Namwon Study

**DOI:** 10.2188/jea.JE20130032

**Published:** 2014-01-05

**Authors:** Sun-Seog Kweon, Min-Ho Shin, Hae-Sung Nam, Seul-Ki Jeong, Kyeong-Soo Park, Jin-Su Choi, Seong-Woo Choi, Hye-Yeon Kim, Gyung-Jae Oh, Young-Hoon Lee

**Affiliations:** 1 Department of Preventive Medicine, Chonnam National University Medical School, Gwangju, Republic of Korea; 2 Jeonnam Regional Cancer Center, Chonnam National University Hwasun Hospital, Hwasun, Republic of Korea; 3 Department of Preventive Medicine, Chungnam National University College of Medicine, Daejeon, Republic of Korea; 4 Department of Neurology & Research Institute of Clinical Medicine, Chonbuk National University Medical School-Chonbuk National University Hospital, Jeonju, Republic of Korea; 5 Department of Preventive Medicine, Seonam University College of Medicine, Namwon, Republic of Korea; 6 Department of Preventive Medicine, Chosun University Medical School, Gwangju, Republic of Korea; 7 Department of Preventive Medicine and Institute of Wonkwang Medical Science, Wonkwang University School of Medicine, Iksan, Republic of Korea; 8 Regional Cardiocerebrovascular Center, Wonkwang University Hospital, Iksan, Republic of Korea

**Keywords:** γ-glutamyltransferase, arterial stiffness, brachial–ankle pulse wave velocity

## Abstract

**Background:**

The results of epidemiologic studies of the association between serum γ-glutamyltransferase (GGT) and brachial–ankle pulse wave velocity (baPWV) have been inconsistent. We examined the association between serum GGT and arterial stiffness in a general population of Korean adults.

**Methods:**

The study population consisted of 6314 community-dwelling Koreans who participated in the baseline survey of the Namwon Study. We analyzed sex-specific association between serum GGT and arterial stiffness, as measured by baPWV.

**Results:**

There was a significant progressive increase in age-adjusted mean baPWV across quartiles of GGT in both sexes. In fully adjusted analysis, as compared with the lowest quartile, the odds ratios (95% CI) for high baPWV (ie, sex-specific fifth quintile) were 1.51 (1.03–2.23), 1.82 (1.22–2.72), and 2.80 (1.79–4.40) among men (*P*-trend <0.001), and 1.11 (0.81–1.52), 1.29 (0.94–1.76), and 1.47 (1.04–2.08) among women (*P*-trend <0.001), for the second, third, and fourth quartiles of GGT, respectively.

**Conclusions:**

This population-based study examined the dose-response relationship between GGT and arterial stiffness as measured by baPWV in both sexes. The association between GGT and arterial stiffness was stronger among men. Additional longitudinal studies are needed to examine the relationship between GGT and arterial stiffness and clarify the mechanism underlying the association.

## INTRODUCTION

Serum γ-glutamyltransferase (GGT) is used as a clinical marker of alcohol consumption and liver disease.^1^ Serum GGT activity is also a marker of oxidative stress^2^ and is associated with cardiovascular events and death.^3^^–^^6^

Pulse wave velocity (PWV) is an index of arterial stiffness and atherosclerotic vascular damage and is also closely associated with cardiovascular events and death.^7^^–^^10^ Brachial–ankle pulse wave velocity (baPWV) is commonly used in epidemiologic and clinical research as a surrogate marker of arterial stiffness and atherosclerosis. Epidemiologic evidence indicates that baPWV, which reflects stiffness of both central and peripheral arteries, is an independent predictor of cardiovascular disease.^11^^–^^13^

A few epidemiologic studies examined the association between serum GGT and arterial stiffness, but the findings were not consistent.^14^^–^^17^ Because several studies reported a positive association between serum GGT and baPWV only in men, it remains to be determined if the association is sex-specific. Therefore, we evaluated the sex-specific association between serum GGT and arterial stiffness, as determined by baPWV, in a large general population.

## METHODS

### Participants

The study population comprised participants in the baseline survey of the Namwon Study, an ongoing population-based prospective study of the prevalence, incidence, and risk factors of cardiovascular disease, osteoporosis, and dementia in a community-dwelling population of Koreans aged 45 to 74 years. A total of 10 667 participants (response rate, 32.3%) were enrolled during the period 2004–2007. Of these, 788 people with a history of coronary heart disease or stroke were excluded, as were 377 with peripheral arterial disease (ankle–brachial index <0.9) and 103 with a history of viral hepatitis B, C, or liver cirrhosis. Another 3085 participants were excluded because of missing data for baPWV (baPWV was not measured in all participants who participated in 2004, *n* = 2035), serum GGT, or other variables. In total, 6314 participants were included in the present analyses (Figure). All participants were fully informed of the study content and gave informed consent for use of their data. This study was approved by the institutional review board of Chonnam National University Hospital.

**Figure.**
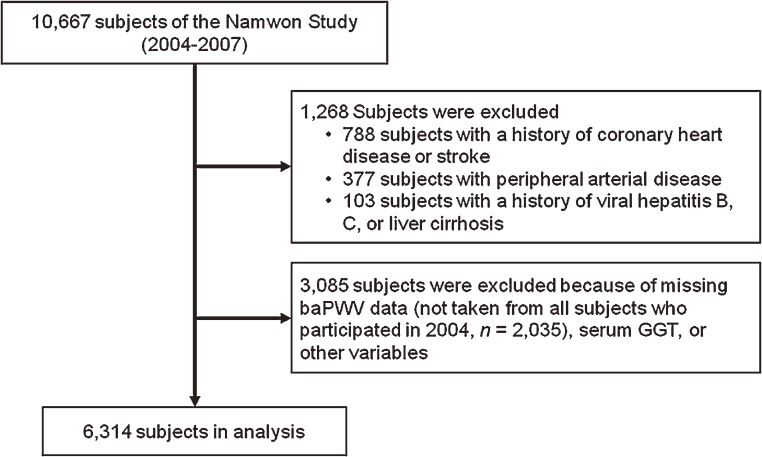
Flow diagram for participant selection.

### Data collection

Information on alcohol consumption, smoking, and the medical history of each participant was collected with a standardized questionnaire by trained research staff. Alcohol intake was assessed using a structured interview, and the amount of ethanol consumed per day was calculated from the average number of alcoholic beverages consumed. Smoking status was classified as never-smoker, former smoker, and current smoker.

All participants underwent a standardized physical examination performed by experienced research staff. Height was measured to the nearest 0.1 cm, and weight was measured to the nearest 0.1 kg, while participants wore light clothing and no shoes. Body mass index (BMI) was calculated as weight in kilograms divided by the height in meters squared. While participants were seated, blood pressure was measured using a mercury sphygmomanometer on the right upper arm after a 5-minute rest. Three consecutive readings of systolic blood pressure (SBP) and diastolic blood pressure (DBP) were recorded at intervals of 1 minute, and the average was used in the analysis.

Blood samples were drawn from an antecubital vein in the morning after a 12-hour overnight fast. Fasting blood glucose (FBG), lipid profile, including total cholesterol (TC), high-density lipoprotein cholesterol (HDL-C), and triglycerides (TG), and liver enzymes, including aspartate aminotransferase (AST), alanine aminotransferase (ALT), and GGT, and uric acid (UA), were analyzed using the enzymatic method with an automatic analyzer (model 7600 chemical analyzer; Hitachi Ltd., Tokyo, Japan). High-sensitivity C-reactive protein (hs-CRP) was measured by latex-enhanced nephelometry using a high-sensitivity assay analyzer (Behring Nephelometer II; Dade-Behring Diagnostics, Marburg, Germany).

baPWV was assessed using a noninvasive, automatic oscillometric device (VP-1000, Colin, Tokyo, Japan). The participants were examined after relaxing in supine position on a bed for at least 5 minutes. Volume waveforms for the brachium and ankle were stored, and the sampling time was 10 seconds, with automatic gain analysis and quality adjustment. The distance between baPWV sampling points was calculated automatically according to the height of the participant. The path lengths from the suprasternal notch to the ankle and those to the brachium were obtained from superficial measurements [La = 0.8129 × height (cm) + 12.328; Lb = 0.2195 × height (cm) − 2.0734], and ΔT was defined as the time interval between the wave front of the brachial waveform and that of the ankle waveform. Finally, the following equation was used to obtain baPWV: baPWV = (La − Lb)/ΔT. We used the mean of the baPWV values for the right and left ankles.

### Statistical analysis

To determine if the association was sex-specific, all analyses were performed separately in men and women. Serum GGT levels were classified into 4 groups, using the 25th, 50th, and 75th percentiles as cut-off points. The sex-specific GGT cut-off points were 19, 29, and 56 U/L, respectively, among men and 11, 14, and 21 U/L among women. Linear trends in baseline characteristics across GGT quartiles were evaluated by analysis of variance and the χ^2^ test. Correlation coefficients were calculated in both sexes to determine associations between GGT and cardiovascular risk factors. Age-adjusted mean baPWV across GGT quartiles was assessed with general linear models. Two logistic regression approaches were used to evaluate the association between GGT and baPWV. High baPWV was defined as the sex-specific fifth quintile of mean baPWV (≥1790.0 cm/s for men and ≥1744.5 cm/s for women). First, in multiple logistic regression analyses we compared odds ratios (ORs) and 95% CIs for high baPWV across GGT quartiles, using the lowest quartile as the reference category. Age, education level, and marital status were controlled in Model 1, and smoking status, daily alcohol consumption, BMI, heart rate, SBP (assessed during baPWV measurement), TC/HDL-C ratio, TG, FBG, antihypertensive medication, antidiabetic medication, UA, AST, and ALT were further adjusted for in Model 2. White blood cells (WBC) and hs-CRP were further adjusted for in Model 3. Second, we estimated the OR for high baPWV based on log-unit changes in GGT. Covariates were controlled for in the same manner as above (Models 1 to 3). Linear trends were evaluated by multiple logistic regression using the GGT categories as a continuous variable. All statistical analyses were performed using SPSS version 18.0 (SPSS Inc., Chicago, IL, USA). A *P* value less than 0.05 was considered to indicate statistical significance.

## RESULTS

### Study population characteristics

Of the 6314 participants analyzed, 2454 (38.9%) were male and 3860 (61.1%) were female. The mean age of participants was 61.3 ± 8.0 years (61.9 ± 7.8 years for men and 60.9 ± 8.1 years for women). Median GGT (interquartile range) was 19 U/L (13–32 U/L) in both sexes, 30 U/L (19–56 U/L) in men, and 15 U/L (12–21 U/L) in women (*P* < 0.001). The baseline characteristics according to GGT quartile are summarized in Table 1 for men and Table 2 for women. Among men, smoking, alcohol consumption, BMI, waist circumference, heart rate, SBP, DBP, antihypertensive medication, FBG, antidiabetic medication, TC, HDL-C, TG, UA, AST, ALT, WBC, hs-CRP, and baPWV were positively associated with GGT quartile, and age was inversely associated with GGT quartile. Among women, smoking, alcohol consumption, BMI, waist circumference, heart rate, SBP, DBP, antihypertensive medication, FBG, antidiabetic medication, TC, TG, UA, AST, ALT, WBC, hs-CRP, and baPWV were positively associated with GGT quartile.

**Table 1.** Baseline characteristics of male participants by quartile of serum γ-glutamyltransferase level (*n* = 2454)

	Quartile 1 [≤19 U/L] (*n* = 640)	Quartile 2 [20–29 U/L] (*n* = 567)	Quartile 3 [30–56 U/L] (*n* = 634)	Quartile 4 [≥57 U/L] (*n* = 613)	*P* for trend
Age, years	63.7 ± 7.9	62.6 ± 7.7	61.0 ± 7.6	60.4 ± 7.7	<0.001
Education level					0.170
≤6 years	377 (58.9)	309 (54.5)	351 (55.4)	375 (61.2)	
7–12 years	223 (34.8)	215 (37.9)	242 (38.2)	214 (34.9)	
≥13 years	40 (6.3)	43 (7.6)	41 (6.4)	24 (3.9)	
Marital status					0.499
Married	598 (93.4)	535 (94.4)	588 (92.7)	582 (94.9)	
Others^a^	42 (6.6)	32 (5.6)	46 (7.3)	31 (5.1)	
Smoking status					<0.001
Never	161 (25.2)	140 (24.7)	129 (20.3)	99 (16.2)	
Former	282 (44.1)	262 (46.2)	312 (49.3)	287 (46.8)	
Current	197 (30.7)	165 (29.1)	193 (30.4)	227 (37.0)	
Alcohol drinking					<0.001
Never	211 (33.0)	119 (21.0)	68 (10.7)	24 (3.9)	
Former	139 (21.7)	114 (20.1)	72 (11.4)	36 (5.9)	
Current	290 (45.3)	334 (58.9)	494 (77.9)	553 (90.2)	
Antihypertensive medication					0.002
No	549 (85.8)	435 (76.7)	490 (77.3)	481 (78.5)	
Yes	91 (14.2)	132 (23.3)	144 (22.7)	132 (21.5)	
Antidiabetic medication					0.035
No	603 (94.2)	519 (91.5)	564 (89.0)	562 (91.7)	
Yes	37 (5.8)	48 (8.5)	70 (11.0)	51 (8.3)	
Alcohol consumption, drinks/day	0.55 ± 1.38	1.25 ± 2.33	2.41 ± 3.35	4.19 ± 4.28	<0.001
Body mass index, kg/m^2^	23.0 ± 2.6	24.1 ± 2.8	24.5 ± 2.6	24.5 ± 2.9	<0.001
Waist circumference, cm	82.2 ± 7.7	85.3 ± 7.8	86.5 ± 7.0	87.2 ± 7.5	<0.001
Heart rate, beats/30 s	34.2 ± 5.0	34.3 ± 5.4	34.9 ± 5.4	35.7 ± 5.9	<0.001
Systolic blood pressure, mm Hg^b^	125.7 ± 15.5	127.8 ± 16.0	130.2 ± 16.1	132.3 ± 16.3	<0.001
Systolic blood pressure, mm Hg	122.0 ± 16.1	124.2 ± 15.8	126.9 ± 16.3	129.8 ± 17.2	<0.001
Diastolic blood pressure, mm Hg	78.1 ± 9.0	80.1 ± 9.5	82.4 ± 9.6	82.9 ± 9.9	<0.001
Fasting blood glucose, mg/dl	101.4 ± 17.4	107.0 ± 23.8	111.3 ± 29.5	114.8 ± 29.6	<0.001
Total cholesterol, mg/dl	174.0 ± 31.2	181.7 ± 32.2	186.6 ± 34.1	185.4 ± 38.1	<0.001
HDL cholesterol, mg/dl	46.4 ± 11.2	45.2 ± 12.0	45.9 ± 11.6	49.2 ± 12.7	<0.001
Triglycerides, mg/dl	99 (71–145)	124 (85–177)	148 (100–226)	188 (117–284)	<0.001
Uric acid, mg/dl	4.9 ± 1.2	5.3 ± 2.7	5.5 ± 3.0	5.9 ± 1.5	<0.001
Aspartate aminotransferase, U/L	22.5 ± 6.0	24.1 ± 7.3	27.0 ± 9.1	40.1 ± 46.4	<0.001
Alanine aminotransferase, U/L	18.1 ± 7.1	22.2 ± 10.2	27.4 ± 15.6	38.5 ± 24.8	<0.001
White blood cells, 10^3^/µL	6.3 ± 1.7	6.7 ± 1.8	6.9 ± 2.0	6.9 ± 1.8	<0.001
hs-CRP, mg/dl	0.05 (0.02–0.14)	0.07 (0.03–0.17)	0.08 (0.04–0.19)	0.10 (0.05–0.22)	<0.001
baPWV, cm/s	1514.9 ± 280.0	1552.8 ± 294.4	1575.8 ± 307.5	1649.6 ± 335.9	<0.001

Data are expressed as mean ± SD, median (interquartile range) for skewed variables, or *n* (%).

Abbreviations: HDL, high-density lipoprotein; hs-CRP, high-sensitivity C-reactive protein; baPWV, brachial–ankle pulse wave velocity.

^a^Unmarried, divorced, separated, or widowed.

^b^Measured during baPWV measurement.

**Table 2.** Baseline characteristics of female participants by quartile of serum γ-glutamyltransferase level (*n* = 3860)

	Quartile 1 [≤11 U/L] (*n* = 928)	Quartile 2 [12–14 U/L] (*n* = 911)	Quartile 3 [15–21 U/L] (*n* = 1059)	Quartile 4 [≥22 U/L] (*n* = 962)	*P* for trend
Age, years	60.5 ± 8.6	61.3 ± 8.1	60.8 ± 8.1	60.8 ± 7.4	0.760
Education level					0.508
≤6 years	754 (81.2)	759 (83.3)	884 (83.5)	797 (82.9)	
7–12 years	165 (17.8)	139 (15.3)	159 (15.0)	153 (15.9)	
≥13 years	9 (1.0)	13 (1.4)	16 (1.5)	12 (1.2)	
Marital status					0.471
Married	698 (75.2)	670 (73.5)	772 (72.9)	711 (73.9)	
Others^a^	230 (24.8)	241 (26.5)	287 (27.1)	251 (26.1)	
Smoking status					0.035
Never	885 (95.4)	870 (95.5)	1003 (94.7)	901 (93.7)	
Former	16 (1.7)	14 (1.5)	21 (2.0)	15 (1.6)	
Current	27 (2.9)	27 (3.0)	35 (3.3)	46 (4.7)	
Alcohol drinking					<0.001
Never	566 (61.0)	520 (57.1)	548 (51.8)	477 (49.5)	
Former	37 (4.0)	46 (5.0)	66 (6.2)	67 (7.0)	
Current	325 (35.0)	345 (37.9)	445 (42.0)	418 (43.5)	
Antihypertensive medication					<0.001
No	790 (85.1)	744 (81.7)	796 (75.2)	682 (70.9)	
Yes	138 (14.9)	167 (18.3)	263 (24.8)	280 (29.1)	
Antidiabetic medication					<0.001
No	897 (96.7)	866 (95.1)	989 (93.4)	868 (90.2)	
Yes	31 (3.3)	45 (4.9)	70 (6.6)	94 (9.8)	
Alcohol consumption, drinks/day	0.07 ± 0.29	0.13 ± 0.81	0.15 ± 0.61	0.22 ± 0.75	<0.001
Body mass index, kg/m^2^	23.4 ± 2.8	24.2 ± 3.0	24.9 ± 3.0	25.5 ± 3.2	<0.001
Waist circumference, cm	83.5 ± 8.1	85.2 ± 8.5	87.3 ± 8.2	88.8 ± 8.3	<0.001
Heart rate, beats/30 s	35.0 ± 4.9	35.2 ± 5.2	35.6 ± 5.2	36.4 ± 5.8	<0.001
Systolic blood pressure, mm Hg^b^	122.8 ± 17.3	125.6 ± 18.3	129.0 ± 18.6	130.4 ± 17.6	<0.001
Systolic blood pressure, mm Hg	119.7 ± 17.5	122.7 ± 18.7	126.0 ± 18.6	126.4 ± 18.8	<0.001
Diastolic blood pressure, mm Hg	77.2 ± 10.5	78.6 ± 10.3	80.2 ± 9.7	81.0 ± 10.5	<0.001
Fasting blood glucose, mg/dl	97.3 ± 19.2	100.0 ± 18.6	103.3 ± 20.9	108.4 ± 24.9	<0.001
Total cholesterol, mg/dl	178.9 ± 32.8	188.7 ± 32.5	197.4 ± 35.2	204.8 ± 38.6	<0.001
HDL cholesterol, mg/dl	48.7 ± 11.7	48.7 ± 12.0	48.1 ± 11.4	48.5 ± 11.9	0.396
Triglycerides, mg/dl	105 (73–146)	120 (83–173)	140 (98–200)	155 (106–226)	<0.001
Uric acid, mg/dl	3.7 ± 0.9	3.8 ± 0.9	4.1 ± 1.0	4.3 ± 1.1	<0.001
Aspartate aminotransferase, U/L	21.1 ± 5.2	21.8 ± 5.8	22.7 ± 7.2	29.8 ± 23.5	<0.001
Alanine aminotransferase, U/L	14.7 ± 6.3	16.4 ± 6.2	19.5 ± 8.7	29.6 ± 24.9	<0.001
White blood cells, 10^3^/µL	5.5 ± 1.4	5.8 ± 1.6	6.2 ± 1.6	6.5 ± 1.9	<0.001
hs-CRP, mg/dl	0.04 (0.02–0.08)	0.05 (0.02–0.11)	0.06 (0.03–0.15)	0.09 (0.04–0.19)	<0.001
baPWV, cm/s	1465.7 ± 287.6	1507.4 ± 311.0	1556.0 ± 318.7	1582.7 ± 327.8	<0.001

Data are expressed as mean ± SD, median (interquartile range) for skewed variables, or *n* (%).

Abbreviations: HDL, high-density lipoprotein; hs-CRP, high-sensitivity C-reactive protein; baPWV, brachial–ankle pulse wave velocity.

^a^Unmarried, divorced, separated, or widowed.

^b^Measured during baPWV measurement.

### Correlation with log-transformed GGT

Serum GGT was weakly inversely correlated with age in men but was not significantly correlated with age in women. Weak to moderate correlations between GGT and liver enzymes (AST and ALT) were observed among men (*r* = 0.376 and 0.511, respectively) and women (*r* = 0.338 and 0.451). Alcohol consumption was moderately correlated with GGT among men (*r* = 0.433) but weakly associated with GGT among women (*r* = 0.117). Serum GGT was weakly correlated with baPWV (*r* = 0.183 in men, *r* = 0.136 in women; Table 3).

**Table 3.** Correlations between serum log-transformed γ-glutamyltransferase level and covariates

	Men		Women	
	
	*r*^a^	*P*-value	*r*^a^	*P*-value
Age	−0.132	<0.001	−0.001	0.940
Alcohol consumption	0.433	<0.001	0.117	<0.001
Body mass index	0.140	<0.001	0.205	<0.001
Waist circumference	0.189	<0.001	0.196	<0.001
Systolic blood pressure	0.182	<0.001	0.104	<0.001
Diastolic blood pressure	0.170	<0.001	0.114	<0.001
Fasting blood glucose	0.210	<0.001	0.187	<0.001
Total cholesterol	0.102	<0.001	0.229	<0.001
HDL cholesterol	0.102	<0.001	0.022	0.169
Triglycerides (log)	0.367	<0.001	0.235	<0.001
Uric acid	0.170	<0.001	0.222	<0.001
Aspartate aminotransferase	0.376	<0.001	0.338	<0.001
Alanine aminotransferase	0.511	<0.001	0.451	<0.001
White blood cells	0.101	<0.001	0.183	<0.001
High-sensitivity C-reactive protein (log)	0.158	<0.001	0.254	<0.001
Brachial–ankle pulse wave velocity	0.183	<0.001	0.136	<0.001

^a^Pearson correlation coefficients.

### Mean baPWV across GGT quartiles

Among men, age-adjusted mean (95% CI) baPWV values for the first, second, third, and fourth GGT quartiles were 1484.3 (1462.9–1505.8), 1541.2 (1518.5–1563.9), 1591.6 (1570.2–1613.1), and 1675.9 (1654.0–1697.8) cm/s, respectively, showing a clear dose-response relationship. Among women, the respective values were 1471.4 (1453.4–1489.3), 1500.1 (1482.0–1518.2), 1556.3 (1539.5–1573.1), and 1583.7 (1566.1–1601.4) cm/s. Mean baPWV showed a significant progressive increase across GGT quartiles among men and women (*P* < 0.001 for both; data not shown).

### Relationship between GGT and high baPWV

The prevalence and ORs for high baPWV, defined as the fifth quintile of baPWV across the GGT quintiles, are shown in Table 4. The prevalence of abnormal baPWV showed a significant progressive increase across GGT quintiles among men and women (*P* < 0.001 for both). In model 1, the age-adjusted ORs (95% CI) for high baPWV were 1.83 (1.31–2.55), 2.71 (1.95–3.75), and 5.30 (3.83–7.34) among men, and 1.33 (1.01–1.74), 2.01 (1.55–2.59), and 2.62 (2.03–3.39) among women, for the second, third, and fourth quartiles of GGT, respectively, as compared with the lowest quartile (*P*-trend <0.001 in both sexes). Additional adjustment for established cardiovascular risk factors (model 2) attenuated these relationships, but GGT remained an independent risk factor for high baPWV in both sexes. After further adjustment for WBC and hs-CRP (model 3), the ORs (95% CI) for high baPWV also increased stepwise across GGT quartiles: 1.51 (1.03–2.23), 1.82 (1.22–2.72), and 2.80 (1.79–4.40) among men (*P*-trend <0.001), and 1.11 (0.81–1.52), 1.29 (0.94–1.76), and 1.47 (1.04–2.08) among women (*P*-trend <0.001), respectively. The ORs for high baPWV according to GGT quartile were higher among men than among women.

**Table 4.** Prevalences and odds ratios (95% CI) for high brachial–ankle pulse wave velocity^a^ by quartile of serum γ-glutamyltransferase level

	Quartile 1	Quartile 2	Quartile 3	Quartile 4	*P* for trend
Men (*n* = 2454)					
*Prevalence (%)*	*13.1*	*18.3*	*20.0*	*28.4*	<0.001
Model 1	1.00	1.83 (1.31–2.55)	2.71 (1.95–3.75)	5.30 (3.83–7.34)	<0.001
Model 2	1.00	1.54 (1.04–2.26)	1.88 (1.26–2.79)	2.91 (1.87–4.54)	<0.001
Model 3	1.00	1.51 (1.03–2.23)	1.82 (1.22–2.72)	2.80 (1.79–4.40)	<0.001
Women (*n* = 3860)					
*Prevalence (%)*	*13.8*	*17.7*	*22.5*	*25.6*	<0.001
Model 1	1.00	1.33 (1.01–1.74)	2.01 (1.55–2.59)	2.62 (2.03–3.39)	<0.001
Model 2	1.00	1.12 (0.82–1.53)	1.31 (0.97–1.79)	1.52 (1.08–2.13)	<0.001
Model 3	1.00	1.11 (0.81–1.52)	1.29 (0.94–1.76)	1.47 (1.04–2.08)	<0.001

^a^High brachial–ankle pulse wave velocity was defined as the fifth quintile (≥1790.0 cm/s for males and ≥1744.5 cm/s for females).

Model 1: adjusted for age, education level, and marital status.

Model 2: adjusted for model 1 covariates plus smoking status, daily alcohol consumption, body mass index, heart rate, systolic blood pressure (measured during baPWV measurement), total/high-density lipoprotein cholesterol ratio, log-transformed triglycerides, fasting glucose, antihypertensive medication, antidiabetic medication, uric acid, aspartate aminotransferase, and alanine aminotransferase.

Model 3: adjusted for model 2 covariates plus white blood cells and log-transformed high-sensitivity C-reactive protein.

R^2^_N_ (Nagelkerke R-squared) values were 0.246, 0.471, and 0.472 in Models 1 to 3, respectively, among men and 0.239, 0.489, and 0.489 among women.

The results of the Omnibus Test of Model Coefficients were <0.001, <0.001, and <0.001 for Models 1 to 3, respectively, among men and <0.001, <0.001, and <0.001 among women. The results of the Hosmer-Lemeshow test were 0.257, 0.331, and 0.187 in Models 1 to 3, respectively, among men and 0.276, 0.788, and 0.802 among women.

### Relationships between log-unit change in GGT and high baPWV

In model 1, the OR (95% CI) for high baPWV per log-unit increase of GGT was 2.04 (1.79–2.33) for men and 1.73 (1.48–2.01) for women. Further adjustment for other variables (models 2 and 3) slightly attenuated these associations, but GGT remained a significant risk factor for high baPWV among men (OR, 1.60; 95% CI, 1.29–1.98) and women (1.35, 1.07–1.69) in the fully adjusted model (Table 5).

**Table 5.** Odds ratios (95% CI) for high brachial–ankle pulse wave velocity^a^ per log-unit increase in γ-glutamyltransferase level

	Men	Women
Model 1	2.04 (1.79–2.33)	1.73 (1.48–2.01)
Model 2	1.62 (1.32–2.00)	1.37 (1.10–1.72)
Model 3	1.60 (1.29–1.98)	1.35 (1.07–1.69)

^a^High brachial–ankle pulse wave velocity was defined as the fifth quintile (≥1790.0 cm/s for men and ≥1744.5 cm/s for women).

Model 1: adjusted for age, education level, and marital status.

Model 2: adjusted for model 1 covariates plus smoking status, daily alcohol consumption, body mass index, heart rate, systolic blood pressure (measured during baPWV measurement), total/high-density lipoprotein cholesterol ratio, log-transformed triglycerides, fasting glucose, antihypertensive medication, antidiabetic medication, uric acid, aspartate aminotransferase, and alanine aminotransferase.

Model 3: adjusted for model 2 covariates plus white blood cells and log-transformed high-sensitivity C-reactive protein.

R^2^_N_ (Nagelkerke R-squared) values were 0.248, 0.450, and 0.451 in Models 1 to 3, respectively, among men and 0.232, 0.452, and 0.452 among women.

The results of the Omnibus Test of Model Coefficients were <0.001, <0.001, and <0.001 for Models 1 to 3, respectively, among men and <0.001, <0.001, and <0.001 among women. The results of the Hosmer-Lemeshow test were 0.387, 0.185, and 0.573 in Models 1 to 3, respectively, among men and 0.203, 0.954, and 0.927 among women.

## DISCUSSION

We investigated the association between serum GGT and arterial stiffness in community-dwelling middle-aged and older Korean adults. After adjusting for established cardiovascular risk factors, a significant dose-response association between serum GGT and high baPWV was identified among men and women. Additionally, the association between GGT and arterial stiffness was stronger among men than among women.

Serum GGT is used as an indicator of excessive alcohol consumption and liver disease in clinical practice,^1^^,^^2^ but several studies noted an association between GGT and cardiovascular risk factors after controlling for alcohol intake.^18^^–^^20^ Emerging epidemiologic evidence shows a significant association between serum GGT and cardiovascular disease.^3^^–^^6^ A recent study reported that GGT may be directly involved in plaque progression and atherogenesis,^21^^,^^22^ suggesting a role for GGT in the pathogenesis of cardiovascular disease.

Aortic PWV, an index of arterial stiffness and atherosclerotic vascular damage, is an independent predictor of cardiovascular morbidity and mortality.^7^^–^^10^ Recent studies have shown that baPWV is closely correlated with invasive aortic PWV, which is the gold standard measure of arterial stiffness.^23^^–^^25^ baPWV measurement is simple, quick, and noninvasive and thus is widely used as a surrogate marker of arterial stiffness and atherosclerosis in community settings and by primary care physicians.^11^^,^^12^ Recent evidence has shown that baPWV is an independent predictor of cardiovascular disease.^11^^–^^13^

A sex difference in the association between serum GGT and PWV was reported in prior epidemiologic studies.^16^^,^^17^ GGT was found to be independently associated with increased baPWV only in men, although the association was very weak (OR, 1.004; 95% CI, 1.001–1.007).^17^ In a study using baPWV Saijo et al reported that GGT was significantly associated with increased arterial stiffness in men but not in women.^16^ Plausible reasons cited for the lack of an association among women included small sample size, overadjustment, and lower GGT and baPWV levels among women as compared with men.^16^ However, 2 recent epidemiologic studies reported a significant association between serum GGT and PWV in both sexes.^14^^,^^15^ The multivariate-adjusted ORs for the highest versus the lowest GGT quartiles were 1.63 (95% CI, 1.21–2.20) in men and 1.56 (1.08–2.27) in women, indicating no sex difference in the association between serum GGT and baPWV.^14^ A recent study found that serum GGT was independently associated with increased peripheral PWV in both sexes: among participants in the highest GGT tertile, the OR (95% CI) for high peripheral PWV was 1.87 (1.31–2.62) for men and 2.41 (1.39–4.62) for women.^15^ Although the present participants were older than those in other studies,^14^^,^^15^ serum GGT was positively associated with high baPWV among men and women, as was the case in other recent studies. The ORs for high baPWV were higher among men than among women; however, we found no sex difference in the association.

Although the association between serum GGT and baPWV was slightly attenuated after controlling for inflammatory markers such as WBC and hs-CRP (model 3), it remained significant in both sexes, which suggests that inflammation partially mediates the relationship between serum GGT and arterial stiffness. Additionally, the noninflammatory pathway might still mediate the link between serum GGT and arterial stiffness. Although the biological mechanism underlying the association between GGT and arterial stiffness is not completely understood, oxidative stress may be involved. GGT is an enzyme responsible for both extracellular catabolism of the antioxidant glutathione and depletion of glutathione due to free radical production, which subsequently elevates serum GGT activity.^2^^,^^26^ Increased GGT activity might be an early, sensitive response to oxidative stress, and people with increased oxidative stress have increased serum concentrations of GGT.^27^ PWV is significantly correlated with oxidative stress^28^ and is an independent predictor of cardiovascular disease^8^^,^^9^; therefore, oxidative stress may mediate the link between GGT and arterial stiffness.

This study had limitations. First, due to its cross-sectional design, we cannot make causal inferences between serum GGT and baPWV. Second, although education level and marital status were controlled for in the analysis, we did not fully adjust for other socioeconomic factors that may be important risk factors for cardiovascular disease and atherosclerosis. Despite these limitations, our study had strengths. First, daily alcohol consumption, calculated using standard drinks per day, and liver enzymes, including AST, ALT, and GGT, were adjusted for in the analysis. Second, UA, WBC, and hs-CRP were adjusted for to investigate the independent association between serum GGT and baPWV after adjusting for chronic inflammatory markers.

In conclusion, this population-based study identified a dose-response relationship between serum GGT and arterial stiffness, as measured by baPWV, in men and women. The association between serum GGT and arterial stiffness was stronger among men than among women. A prospective cohort study of the association between GGT and baPWV with repeated measures is necessary to establish a causal relationship between serum GGT and arterial stiffness and to clarify the underlying mechanism.
